# Sexually transmitted infection and teenage pregnancy in adolescents having parents with schizophrenia: a retrospective cohort study of 64,350 participants

**DOI:** 10.1007/s00787-024-02470-2

**Published:** 2024-05-24

**Authors:** Ju-Wei Hsu, Li-Chi Chen, Kai-Lin Huang, Shih-Jen Tsai, Ya-Mei Bai, Tung-Ping Su, Tzeng-Ji Chen, Mu-Hong Chen

**Affiliations:** 1 Department of Psychiatry, Taipei Veterans General Hospital, Taipei, Taiwan; 2 Department of Psychiatry, College of Medicine, National Yang Ming Chiao Tung University, Taipei, Taiwan; 3 Department of Family Medicine, Taipei Veterans General Hospital, Taipei, Taiwan; 4 Institute of Hospital and Health Care Administration, National Yang Ming Chiao Tung University, Taipei, Taiwan; 5 Department of Psychiatry, General Cheng Hsin Hospital, Taipei, Taiwan; 6 Department of Family Medicine, Hsinchu Branch, Taipei Veterans General Hospital, Hsinchu, Taiwan

**Keywords:** Offspring, Schizophrenia, Sexually transmitted infections, Teenage pregnancy

## Abstract

**Background:**

The risks of sexually transmitted infections (STIs) and teenage pregnancy in the offspring of parents with schizophrenia remain unknown.

**Methods:**

From the Taiwan National Health Insurance Research Database, 5,850 individuals born between 1980 and 1999 having any parent with schizophrenia and 58,500 age-, sex-, income- and residence-matched controls without parents with severe mental disorders were enrolled in 1996 or on their birthdate and followed up to the end of 2011. Those who contracted any STI or became pregnant in adolescence during the follow-up period were identified.

**Results:**

Cox regression analyses demonstrated that offspring of parents with schizophrenia (hazard ratio [HR]: 1.21, 95% confidence interval [CI]: 1.02–1.44), especially daughters (HR: 1.30, 95% CI: 1.06–1.58), were more likely to contract any STI later in life than the control comparisons. In addition, daughters of parents with schizophrenia had an elevated risk of being pregnant in their adolescence (HR: 1.47, 95% CI: 1.29–1.67) compared with those having no parents with severe mental disorders.

**Discussion:**

The positive relationship between parental schizophrenia and offspring STIs and teenage pregnancy necessitates clinicians and public health officers to closely monitor the sexual health in the offspring of parents with schizophrenia so that optimal and prompt preventive measures can be taken in the at-risk group.

Sexually transmitted infection (STI) and teenage pregnancy greatly affect the mental and physical well-being of the adolescent population not only in developing countries but also in developed countries [1, 2]. Concerns regarding adolescent sexual and reproductive health and rights have significantly increased since the 1994 International Conference on Population and Development [1]. In Taiwan, the age-specific fertility rate for adolescent girls peaked at 18 per 1,000 in 1994 and gradually declined to 4 per 1,000 in 2015, which is still higher than in other Asian countries such as South Korea (~ 2.8‰) [3]. Based on the data from Taiwan Centers for Disease Control, the incidence rates of STIs, including syphilis, gonorrhea, and HIV, in teenagers aged between 15 and 19 years have considerably increased in the last decade.

Evidence has indicated significantly increased risks of various mental disorders and conditions, including attention deficit hyperactivity disorder (ADHD), schizophrenia, bipolar disorder, major depressive disorder, impaired cognitive function, and deficits in behavioral control and emotional regulation, in the offspring of parents with schizophrenia [4–6]. Our previous study involving 77,547 individuals with a parent with schizophrenia revealed that the offspring of parents with schizophrenia were more likely to be diagnosed with ADHD, schizophrenia, bipolar disorder, and major depressive disorder later in life than the general population [4]. Sandstrom et al. reported that the incidence of attentional problems, oppositional behaviors, and language/thought problems was elevated in the offspring of parents with schizophrenia and further suggested that externalizing problems and cognitive and language difficulties may be shared in a family having individuals with schizophrenia [7]. A study in Denmark of all offspring born between 1980 and 1994 with follow-up to 2008 demonstrated that the risks of psychotic and affective disorders as well as substance use disorder were significantly increased in the offspring of parents with severe mental disorder compared with those having mentally healthy parents [8]. The abovementioned mental conditions, especially ADHD, cognitive dysfunction, and externalizing symptoms, have been associated with risky sexual behaviors, such as unprotected intercourse, multiple sex partners, and illicit drug use [9, 10]. However, no study has assessed the risks of STIs and teenage pregnancy in this minority group with clinically high risks of psychosis.

In this longitudinal follow-up study, we used a large sample based on data from the Taiwan National Health Insurance Research Database of the offspring of parents with schizophrenia to investigate the risks of STIs and teenage pregnancy in the offspring of parents with schizophrenia compared with the control individuals having no parent with any severe mental disorder. We hypothesized that parental schizophrenia would be related to increased risks of STIs and teenage pregnancy in their offspring.

## Methods

### Data source

The Taiwan NHIRD is audited and released by the National Health Research Institute (NHRI) for scientific and study purposes upon the formal application. In current study, we linked three databases together for the analysis. The first is the registry database for all beneficiaries (~ 28,000,000), which was used for the genealogy reconstruction and demographic characteristics based on Chen et al’s and Cheng et al’s methods [4, 11]. Only blood relatives or spouses were qualified to be dependents of the insured patients. With unique personal identifiers, we identified the following family relationship groups, including parents and offspring. In order to ensure the complete data of the father-mother-offspring triad, we only included those who could be linked to both fathers and mothers in our study. In all, 33,485 individuals who were only linked to one of their parents (father or mother) were not included in the present study. The second is the specialized dataset of mental disorders in the NHIRD, which includes all psychiatric medical records of insured patients with mental disorders, including schizophrenia. The third is the Longitudinal Health Insurance Database (LHID), which includes all medical records between 1996 and 2011, including dates of clinical visits, disease diagnoses and prescriptions, of 3,000,000 insured individuals that are randomly selected from entire Taiwanese people (~ 28,000,000), and was used for the identification of study outcomes and related confounding factors. The diagnostic codes used were based on the International Classification of Diseases, 9th Revision, Clinical Modification (ICD-9-CM). The NHIRD has been used extensively in many epidemiologic studies in Taiwan [4, 11–13]. Institutional Review Board of Taipei Veterans General Hospital approved the study protocol and waived the requirement for informed consent since this investigation used de-identified data and no human subjects contact was required.

### Study population

Individuals born between 1980 and 1999 who had any parent with schizophrenia (ICD-9-CM code: 295) were identified as the exposed cohort because the LHID would at least cover a part of adolescence for enrolled individuals. For each case, ten matched controls were randomly selected from the candidates who had no parent with any severe mental disorder (ICD-9-CM codes: 295, 296, 300.4, and 311) based on age (± 1 year), sex, income, and residence. Follow-up duration ranged between Jan 1 1996 or birthdate and Dec 31 2011 or death, which ensures that everyone experienced his or her adolescence in the study period. Income and urbanization levels were used to represent socioeconomic status. Level of urbanization (level 1 to level 5; level 1: most urbanized region; level 5: least urbanized region) was also assessed for our study [14].

### Main outcomes

Two main outcomes were assessed in current study. First, any STI, including HIV (ICD-9-CM codes: 042, V08), syphilis (ICD-9-CM codes: 091–097), genital warts (ICD-9-CM code: 078.11), gonorrhea (ICD-9-CM code: 098), chlamydial infection (ICD-9-CM codes: 078.8, 078.88), and trichomoniasis (ICD-9-CM code: 131), was identified during the follow-up period. Second, teenage pregnancy, which is defined as a pregnancy < 20 years, was also identified in the study period.

### Mental comorbidities

Psychiatric comorbidities of enrolled subjects, including autism spectrum disorder (ICD-9-CM codes: 299.0, 299.8, and 299.9), ADHD (ICD-9-CM code: 314), disruptive behavior disorders (ICD-9-CM codes: 312, 313.81), schizophrenia (ICD-9-CM code: 295), bipolar disorder (ICD-9-CM codes: 296.0, 296.1, 296.4, 296.5, 296.6, 296.7, 296.80, 296.81, 296.89), major depressive disorder (ICD-9-CM codes: 296.2, 296.3, 300.4, and 311), alcohol use disorders (ICD-9-CM codes: 291, 303, and 305.0), substance use disorders (ICD-9-CM codes: 292, 304, 305.2, 305.3, 305.4, 305.5, 305.6, 305.7, 305.8, and 305.9), and intellectual disabilities (ICD-9-CM codes: 317, 318, and 319), were assessed as the confounding factors in our study. All mental disorder diagnoses were given by board-certified psychiatrists at least twice, yielding the improved diagnostic validity [15, 16].

### Statistical analysis

For between-group comparisons, the F test was used for continuous variables and Pearson’s X^2^ test for nominal variables, where appropriate. Cox regression analyses with the adjustment of demographic data (age, sex, income, level of urbanization) and psychiatric comorbidities were performed to calculate the hazard ratios (HRs) with 95% confidence intervals (CIs) of any STI and teenage pregnancy between two groups. Subanalyses stratified by sex were also assessed for the relationship between parental schizophrenia and offspring STI risk. A 2-tailed *P*-value of less than 0.05 was considered statistically significant. All data processing and statistical analyses were performed with Statistical Package for Social Science (SPSS) version 17 software (SPSS Inc.) and Statistical Analysis Software (SAS) version 9.1 (SAS Institute, Cary, NC).

## Results

In all, 5850 subjects having any parent with schizophrenia and 58,500 age-, sex-, income- and residence-matched controls without parents with any severe mental disorder were enrolled in current study (Table 1). Offspring of parents with schizophrenia had higher prevalence of ASD (0.4%vs. 0.2%, *p* = 0.004), ADHD (2.4% vs. 1.4%, *p* < 0.001), disruptive behavior disorder (0.5% vs. 0.2%, *p* < 0.001), schizophrenia (1.9% vs. 0.4%, *p* < 0.001), bipolar disorder (0.9% vs. 0.2%, *p* < 0.001), major depressive disorder (2.3% vs. 1.5%, *p* < 0.001), alcohol use disorder (0.4% vs. 0.2%, *p* = 0.011), substance use disorder (0.8% vs. 0.3%, *p* < 0.001), and intellectual disabilities (1.5% vs. 0.5%, *p* < 0.001) compared with those having no parents with any severe mental disorder (Table 1). Furthermore, increased cumulative incidence of STIs (2.6% vs. 2.1%, *p* = 0.014) and teenage pregnancy (9.7% vs. 6.6%, *p* < 0.001) were noted in the offspring of parents with schizophrenia compared with the control cohort (Table 1).

**Table 1:** Demographic characteristics and incidence of STI and teenage pregnancy in the offspring of parents with schizophrenia and control group

	Offspring of parents with schizophrenia (*n* = 5850)	Control group (*n* = 58,500)	*p*-value
Age at enrollment (years, SD)	6.36 (5.30)	6.34 (5.29)	0.873
Age at study end (years, SD)	23.84 (6.01)	23.84 (5.98)	0.964
Sex (n, %)			> 0.999
Male	3025 (51.7)	30,250 (51.7)	
Female	2825 (48.3)	28,250 (48.3)	
Level of urbanization (n, %)			> 0.999
1 (most urbanized)	1162 (19.9)	11,620 (19.9)	
2	1879 (32.1)	18,790 (32.1)	
3	817 (14.0)	8170 (14.0)	
4	621 (10.6)	6210 (10.6)	
5 (most rural)	1371 (23.4)	13,710 (23.4)	
Income-related insured amount (n, %)			> 0.999
≤ 19,100 NTD/month	1519 (26.0)	15,190 (26.0)	
19,001 ~ 42,000 NTD/month	2365 (40.4)	23,650 (40.4)	
> 42,000 NTD/month	1966 (33.6)	19,660 (33.6)	
Mental comorbidities (n, %)			
ASD	26 (0.4)	137 (0.2)	0.004
ADHD	143 (2.4)	793 (1.4)	< 0.001
Disruptive behavior disorder	31 (0.5)	142 (0.2)	< 0.001
Schizophrenia	109 (1.9)	249 (0.4)	< 0.001
Bipolar disorder	54 (0.9)	134 (0.2)	< 0.001
Major depressive disorder	135 (2.3)	873 (1.5)	< 0.001
Alcohol use disorder	24 (0.4)	132 (0.2)	0.011
Substance use disorder	47 (0.8)	192 (0.3)	< 0.001
Intellectual disabilities	89 (1.5)	275 (0.5)	< 0.001
Cumulative incidence of STI (n, %)	150 (2.6)	1212 (2.1)	0.014
Age at STI (years, SD)	19.65 (6.53)	19.44 (6.45)	0.714
Cumulative incidence of teenage pregnancy# (n, %)	273 (9.7)	1875 (6.6)	< 0.001
Age at pregnancy (years, SD)	17.82 (1.58)	17.78(1.71)	0.665

#: only in female sample

ASD: autism spectrum disorder; ADHD: attention deficit hyperactivity disorder; NTD: new Taiwan dollar; SD: standard deviation; STI: sexually transmitted infection

**Fig. 1:**
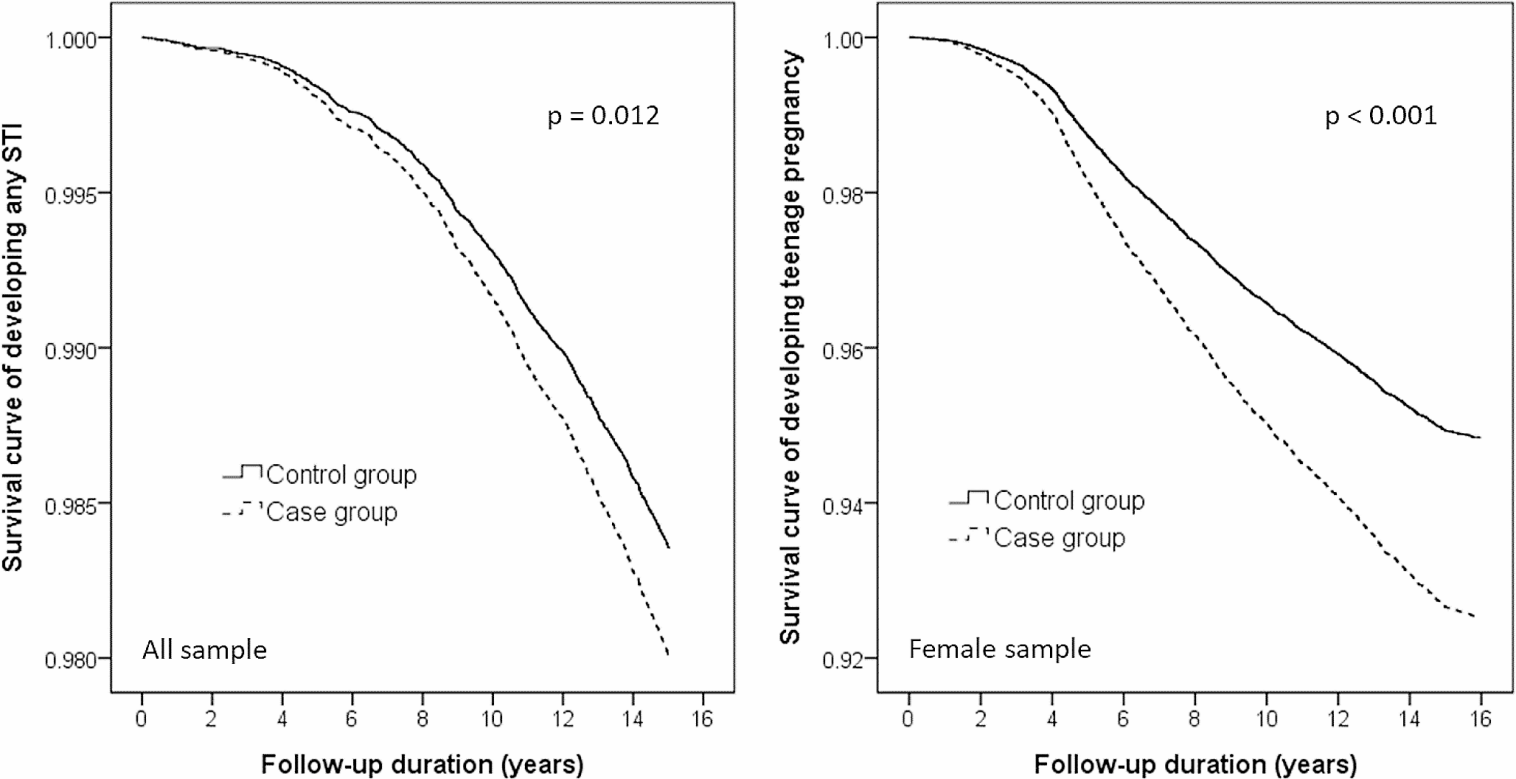
STI and teenage pregnancy risks in the offspring of parents with schizophrenia and control group

Kaplan-Meier survival analyses with log-rank tests revealed significant associations between parental schizophrenia and offspring STIs (*p* = 0.012) and teenage pregnancy (*p* < 0.001) (Fig. 1). Cox regression analyses with full adjustment of demographic data and mental comorbidities showed that offspring of schizophrenia (HR: 1.21, 95% CI: 1.02–1.44), especially daughters (HR: 1.30, 95% CI: 1.06–1.58), were more likely to contract any STI later in life than the control comparisons (Tables 2 and 3). In addition, daughters of parents with schizophrenia had an elevated risk of being pregnant in their adolescent (HR: 1.47, 95% CI: 1.29–1.67) compared with those having no parents with severe mental disorders (Table 3).

**Table 2:** STI risk in the offspring of parents with schizophrenia and control group*

	*n* (%)	HR (95% CI)
All sample		
Offspring of parents with schizophrenia	150 (2.6)	**1.21 (1.02–1.44)**
Control group	1212 (2.1)	1 (ref)
Male sample		
Offspring of parents with schizophrenia	40 (1.3)	1.04 (0.75–1.44)
Control group	377 (1.2)	1 (ref)
Female sample		
Offspring of parents with schizophrenia	110 (3.9)	**1.30 (1.06–1.58)**
Control group	835 (3.0)	1 (ref)

STI: sexually transmitted infection; HR: hazard ratio; CI: confidence interval

* Adjusted by demographic data and mental comorbidities

**Bold type** indicates statistical significance

**Table 3:** Early pregnancy risk in the female offspring of parents with schizophrenia and control group*

	*n* (%)	HR (95% CI)
Female sample		
Offspring of parents with schizophrenia	273 (9.7)	**1.47 (1.29–1.67)**
Control group	1875 (6.6)	1 (ref)

HR: hazard ratio; CI: confidence interval

* Adjusted by demographic data and mental comorbidities

**Bold type** indicates statistical significance

STI: sexually transmitted infection

## Discussion

Our study findings supported the hypothesis that the offspring of parents with schizophrenia, especially daughters, have greater risks of contracting an STI later in life and becoming pregnant in their teenage years than those without parents with severe mental disorders. However, the ages of STIs and teenage pregnancy did not differ between the two groups. Parental schizophrenia was an independent risk factor for offspring STIs and teenage pregnancy after adjustment for offspring mental comorbidities, including ADHD and substance use disorders. However, parental schizophrenia did not accelerate these risks among their offspring.

Neuropsychological evidence suggests the crucial roles of executive function, decision-making, working memory, and impulsivity in risky sexual behaviors, including unprotected sex, early sexual debut, illicit drug use, and having more than one sexual partner [17, 18]. Risky sexual behaviors are the leading cause of subsequent STIs and teenage pregnancy [17, 18]. A community-based adolescent cohort study demonstrated that working memory and impulsivity mediated the relationships between low socioeconomic status and early pubertal maturation and subsequent risks of early sexual initiation and unprotected sex [18]. Studies based on the Iowa Gambling Task and Balloon Analogue Risk Task revealed a significant association between risky sexual behaviors and decision-making and cognitive flexibility as well as reward processing [19, 20]. Hansen et al. examined the relationship between neurocognitive function during response inhibition and frequency of intercourse without a condom among adolescents and found a negative correlation between condom use during the most recent penetrative sex act and dorsolateral prefrontal cortex activity [21]. Xue et al. assessed the insula functioning in 85 sexually active young men who performed an erotic Go/NoGo task in the MRI scanner and revealed that activity in the dorsal anterior insular cortex was negatively correlated with the frequency of condomless intercourse over the past 90 days [22]. Cognitive dysfunction, including deficits in working memory, impulse control, cognitive flexibility, and decision-making, and related impaired brain functioning (i.e., prefrontal cortex and anterior insula) have been regarded as the core neuropathophysiology of schizophrenia and are commonly shared between parents with schizophrenia and their offspring [23, 24].

In addition, offspring psychiatric disorders, especially ADHD, disruptive behavior disorder, psychotic and affective disorders, and substance use disorder, play a crucial role in the association between parental schizophrenia and offspring STIs and teenage pregnancy. Increasing evidence indicates that youth with ADHD, conduct disorder, schizophrenia, and bipolar disorder are more likely to contract an STI later in life compared with those without these mental conditions [25–28]. A naturalistic, longitudinal study of adolescent girls following their first manic episode revealed that 30% of them experienced an unplanned pregnancy at least once during the follow-up period while they were still under the age of 20 years [29]. Goldstein et al. reported that adolescent girls with bipolar disorder and substance use disorder had a significantly greater 12-month prevalence of pregnancy and abortion compared with those without mental illness [30]. Our previous study demonstrated that the risk of teenage pregnancy was significantly increased in adolescents with ADHD compared with those without ADHD and was partially reduced after optimal ADHD medication treatment [31]. Notably, in the current study, we found that parental schizophrenia has an independent role in the risk of offspring STIs and teenage pregnancy regardless of offspring psychiatric disorders.

Previous studies have suggested that adolescents with risky sexual behaviors or teenage pregnancy are more likely to have experienced childhood adversities, including sexual maltreatment, childhood bullying, and childhood neglect, than the controls without those circumstances [32–35]. Ribeiro et al. found that childhood adversities, such as emotional neglect and physical abuse, were related to teenage pregnancy and depression [35]. A Finnish birth cohort study indicated that both bullies and victims had an increased risk of teenage pregnancy [32]. Lehti et al. further reported that bullying by and victimization of the girls themselves, their parents, and their teachers were all associated with teenage pregnancy [32]. Evidence indicated that children of parents with schizophrenia perceived less emotional and physical support from their parents compared with the children of parents without severe mental disorders [36]. Poor or inadequate affective and cognitive parenting is prevalent in families with schizophrenia, which further increases the risk of childhood adversities [37]. However, the complicated association among parental schizophrenia, childhood adversities, and risky sexual behaviors requires further investigation.

Finally, our study only found daughters, but not sons, of parents with schizophrenia had increased risks of subsequent STIs and teenage pregnancy during the follow-up compared with the controls, which may reflect a common clinical scenario that only women would get pregnant and be more likely to visit the obstetrics and gynecology clinic, so women/daughters stood a higher chance to receive clinical attention. This may be a considerable possibility of identification bias in our study. In addition, several studies demonstrated that among emerging adults (aged 18–25 years), women were more knowledgeable about sexual health than men, despite both sexes being not as knowledgeable overall on sexual health topics as expected [38, 39]. The discrepancy in sexual knowledge and attitude between sexes may partially explain our finding.

Several study limitations should be mentioned. First, the cumulative incidence of STIs and teenage pregnancy may be underestimated because only those who sought medical help and treatment could be identified in the database. Additional community-based cohort studies are required to validate our findings. Second, we only included individuals who were linked to both fathers and mothers in the present study, which may underestimate the study findings owing to the exclusion of those who were only linked to fathers or mothers. Third, information on parenting style, family functioning, childhood adversities, and environmental factors was not available in the database. Therefore, we could not investigate the impact of these factors.

In conclusion, the offspring of parents with schizophrenia had increased risks of contracting an STI later in life and becoming pregnant in adolescence compared with the control cohort. The association between parental schizophrenia and offspring STIs as well as teenage pregnancy necessitates that clinicians and public health officers closely monitor the sexual health in the offspring of parents with schizophrenia so that preventive strategies against STIs and teenage pregnancy can be implemented in this minority group with a high clinical risk of psychosis.

## Data Availability

The NHIRD was released and audited by the Department of Health and Bureau of the NHI Program for the purpose of scientific research (https://nhird.nhri.org.tw/). NHIRD can be obtained through the formal application that is regulated by Department of Health and Bureau of the NHI Program.
